# New Organometallic Ru(II) Compounds with Lonidamine Motif as Antitumor Agents

**DOI:** 10.3390/pharmaceutics15051366

**Published:** 2023-04-29

**Authors:** Ilya A. Shutkov, Yulia N. Okulova, Dmitrii M. Mazur, Nikolai A. Melnichuk, Denis A. Babkov, Elena V. Sokolova, Alexander A. Spasov, Elena R. Milaeva, Alexey A. Nazarov

**Affiliations:** 1 Department of Chemistry, M. V. Lomonosov Moscow State University, Leninskie Gory 1/3, 119991 Moscow, Russia; nosovayulia@yahoo.com (Y.N.O.); neodmitrii@gmail.com (D.M.M.); nick.melnichuk@gmail.com (N.A.M.); milaeva@med.chem.msu.ru (E.R.M.); 2 Scientific Center for Innovative Drugs, Volgograd State Medical University, 39 Novorossiyskaya Street, 400087 Volgograd, Russia; denis.a.babkov@gmail.com (D.A.B.); sokolova210795@gmail.com (E.V.S.); aspasov@mail.ru (A.A.S.)

**Keywords:** ruthenium compounds, lonidamine, antiproliferative activity, ligand exchange, mode of action

## Abstract

The combination of one molecule of organic and metal-based fragments that exhibit antitumor activity is a modern approach in the search for new promising drugs. In this work, biologically active ligands based on lonidamine (a selective inhibitor of aerobic glycolysis used in clinical practice) were introduced into the structure of an antitumor organometallic ruthenium scaffold. Resistant to ligand exchange reactions, compounds were prepared by replacing labile ligands with stable ones. Moreover, cationic complexes containing two lonidamine-based ligands were obtained. Antiproliferative activity was studied in vitro by MTT assays. It was shown that the increase in the stability in ligand exchange reactions does not influence cytotoxicity. At the same time, the introduction of the second lonidamine fragment approximately doubles the cytotoxicity of studied complexes. The ability to induce apoptosis and caspase activation in tumour cell MCF7 was studied by employing flow cytometry.

## 1. Introduction

**Figure 1:**
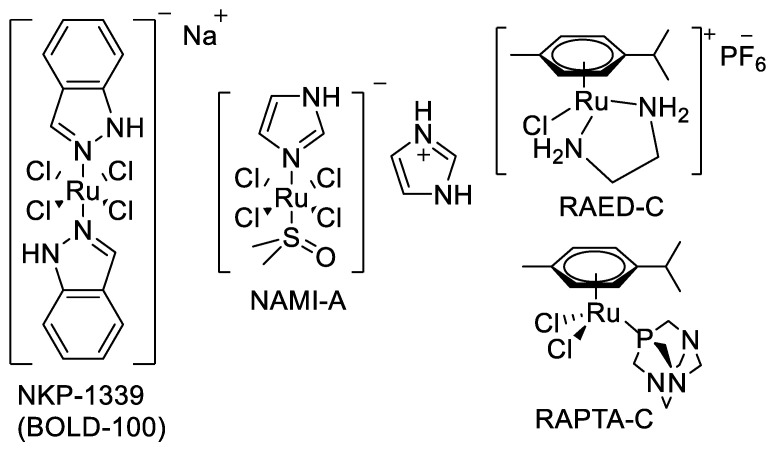
Structures of ruthenium lead compounds.

Platinum-based drugs have been successfully used in clinical practice as anticancer drugs for a long time, but side effects and primary or acquired resistance limited their use [1,2,3,4]. Ruthenium compounds are the most promising replacement due to their unique mode of action; for example, they do not show cross-resistance to platinum drugs and possess relatively low general toxicity in in vivo tests [5,6,7,8,9,10,11,12,13,14]. Two coordination Ru(III) compounds (Figure 1) NAMI-A and NKP-1339 (BOLD-100) were the first to enter into clinical trials [15,16,17,18,19,20,21]. In preclinical trials, it was shown that NAMI-A was less effective against a primary tumour but exhibits activity against metastases [22]. Unfortunately, the compound was found to be insufficiently effective and was withdrawn from clinical trials [23]. BOLD-100 was recently approved by FDA as an orphan drug designated for the treatment of gastric cancer [24]. Organometallic derivatives, such as RAPTA and RAED, come from another promising class of ruthenium antitumor compounds being included in advanced preclinical studies [25,26,27,28,29,30,31,32].

**Figure 2:**
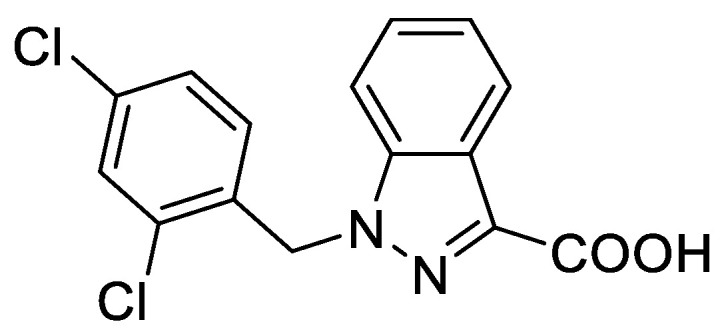
Structure of 1-(2,4-dichlorobenzyl)-1*H*-indazole-3-carboxylic acid (lonidamine).

It is known that the introduction of bioactive organic moieties into the structure of metal-based agents can increase anticancer activity and selectivity due to the interaction with several molecular targets [33,34,35]. One of the most important metabolic features of malignant cells is their increased glycolytic activity known as the Warburg Effect [36,37]. Lonidamine (Figure 2) stimulates lactate production in noncancer cells and reduced glycolysis in their malignant counterparts by inhibiting mitochondrial-associated hexokinase or reprogramming cellular metabolism and mitochondrial function [38,39,40,41]. Lonidamine is widely studied for the treatment of different types of cancer [42,43,44] and is of special interest in the development of dual-acting anticancer compounds.

Previously, lonidamine was introduced into the structures of Pt(IV) complexes [35,45,46] and Ru(II/III) compounds [47,48,49]. The obtained platinum prodrugs and ruthenium twin drugs showed a significantly improved cytotoxicity, superiority to cisplatin and lonidamine, and also some degree of selectivity [35]. The Ru(III) complexes were also shown to be non-competitive thioredoxin reductase inhibitors that effectively induce apoptosis via caspase activation incubation for 24 h. The cytotoxicity of the Ru(III) complexes as well as cellular uptake, apoptosis induction, and thioredoxin reductase inhibition positively correlate with the length of the linker between the ruthenium center and lonidamine moiety [49]. Two organometallic Ru(II) lonidamine conjugates showed promising cytotoxicity on human glioblastoma cell lines and also exhibited a degree of selectivity towards these cells [47].

This work aims to introduce lonidamine-containing ligands into the structure of organometallic Ru(II) compounds to study the antitumor activity and its dependence on the distance between the lonidamine moiety and ruthenium centre, ligand exchange reactions, and the number of lonidamine moieties in the molecule as well as a possible mode of action of cell death via apoptosis induction and caspase activation.

## 2. Materials and Methods

All solvents were purified and degassed before use [50]. Ligands **1–6** were prepared following the published procedure [47,49]. NMR spectra were recorded on a Bruker Avance II 400 spectrometer at room temperature at 400.13 (^1^H) and 100.61 (^13^C{^1^H}) MHz. 2D NMR measurements were carried out using standard pulse programs. Chemical shifts were referenced relative to the solvent signal for ^1^H and ^13^C spectra. Elemental analysis was performed with MicroCube Elementar analyzer. Electrospray ionization (ESI) mass spectra were recorded using a TSQ Endura (Thermo Fisher Scientific, Waltham, MA, USA) instrument. Each analysed compound was dissolved in methanol (HPLC grade) and injected directly into the ionization source through a syringe pump. The spectra were recorded during 30 s in the *m*/*z* range 150–1400 in both positive and negative ionization modes with spray voltage 3.4 and 2.5 kV, correspondingly. The human HCT116 colorectal carcinoma, A549 non-small cell lung carcinoma, MCF7 breast adenocarcinoma and SW480 colon adenocarcinoma cell lines were obtained from the European collection of authenticated cell cultures (ECACC; Salisbury, UK).

### 2.1. Synthesis

**(η^6^-*p*-cymene){*N*-(2-(1*H*-imidazol-1-yl)ethyl)-1-(2,4-dichlorobenzyl)-1*H*-indazole-3-carboxamide}ruthenium(II)-*N* dichloride (7)**


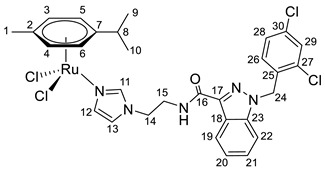


*N*-(2-(1*H*-imidazol-1-yl)ethyl)-1-(2,4-dichlorobenzyl)-1*H*-indazole-3-carboxamide **1** (80 mg, 0.193 mmol) in 5 mL of CH_2_Cl_2_ was added to the dimer (η^6^-*p*-cymene-RuCl_2_)_2_ solution (59 mg, 0.096 mmol) in 5 mL CH_2_Cl_2_. The reaction was stirred for 10 h at room temperature. The reaction mixture was evaporated to 1 mL, and 10 mL of ether and 15 mL of hexane were added. The resulting orange solid was filtered off, washed with hexane, and dried in a vacuum. Yield 70 mg (50%), T_dec_. = 103–105 °C.

^1^H NMR (400.13 MHz, CDCl_3_) δ: 8.33 (d, 1H, J = 8.2 Hz, H19), 7.97 (s, 1H, H11), 7.46–7.27 (m, 6H, H20–22, H29, H12, NH), 7.19 (dd, 1H, J = 8.2, 1.6 Hz, H28), 6.95–6.85 (m, 2H, H13, H26), 5.67 (s, 2H, H24), 5.37 (d, 2H, J = 5.8 Hz, H5, H6), 5.18 (d, 2H, J = 5.8 Hz, H3, H4), 4.14 (t, 2H, J = 4.4 Hz, H14), 3.73 (q, 2H, J = 4.3 Hz, H15), 2.98–2.85 (m, 1H, H8), 2.08 (s, 3H, H1), 1.21 (d, 6H, J = 6.9 Hz, H9, H10).

^13^C{^1^H} NMR (100.61 MHz, CDCl_3_) δ: 162.9 (C16), 141.1 (C17), 140.0 (C23), 137.5 (C11), 134.7 (C25), 133.3 (C30), 132.2 (C27), 132.1 (C29), 130.3 (C26), 129.5 (C12), 127.9 (C28), 127.5 (C21), 123.2 (C19), 122.9 (C18), 122.6 (C20), 120.1 (C13), 109.6 (C22), 102.8 (C7), 97.1 (C2), 82.4 (C5, C6), 81.5 (C3, C4), 50.1 (C24), 47.9 (C14), 39.8 (C15), 30.7 (C8), 22.2 (C9, C10), 18.4 (C1).

Elem. anal. Calc. (%) for C_30_H_31_Cl_4_N_5_ORu: C 50.01, H 4.34, and N 9.72. Found: C 49.64, H 4.22, and N 9.61.

ESI-MS: *m*/*z* 686 [M − Cl]^+^.

**(η^6^-*p*-cymene){*N*-(4-(1*H*-imidazol-1-yl)butyl)-1-(2,4-dichlorobenzyl)-1*H*-indazole-3-carboxamide}ruthenium(II)-*N* dichloride (9)**


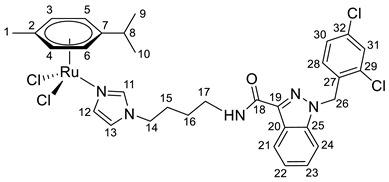


*N*-(4-(1*H*-imidazol-1-yl)butyl)-1-(2,4-dichlorobenzyl)-1*H*-indazole-3-carboxamide **3** (100 mg, 0.22 mmol) in 5 mL CH_2_Cl_2_ was added to the dimer (η^6^-*p*-cymene-RuCl_2_)_2_ solution (69 mg, 0.11 mmol) in 5 mL of CH_2_Cl_2_. The reaction mixture was stirred for 10 h. The solution was evaporated to 1 mL, and 10 mL of ether and 15 mL of hexane were added. The resulting orange precipitate was filtered off, washed with hexane, and dried in a vacuum. Yield 70 mg (41%), T_dec_. = 62–64 °C.

^1^H NMR (400.13 MHz, CDCl_3_) δ: 8.41 (d, 1H, J = 8.1 Hz, H21), 7.93 (s, 1H, H11), 7.49–7.29 (m, 5H, H22–24, H31, H12), 7.18–7.08 (m, 2H, H30, NH), 6.92 (s, 1H, H13), 6.71 (d, 1H, J = 8.3 Hz, H28), 5.69 (s, 2H, H26), 5.44 (d, 2H, J = 5.7 Hz, H5, H6), 5.26 (d, 2H, J = 5.7 Hz, H3, H4), 3.99 (t, 2H, J = 7.1 Hz, H14), 3.56–3.46 (m, 2H, H17), 3.02–2.92 (m, 1H, H8), 1.96–1.82 (m, 2H, H16), 1.72–1.56 (m, 5H, H15, H1), 1.27 (d, 6H, J = 6.9 Hz, H9, H10).

^13^C{^1^H} NMR (100.61 MHz, CDCl_3_) δ: 162.7 (C18), 141.2 (C19), 139.7 (C11), 138.2 (C25), 134.6 (C27), 133.2 (C29/C32), 132.3 (C29/C32), 132.2 (C31), 129.6 (C28), 129.5 (C12), 127.7 (C30), 127.5 (C23), 123.1 (C21), 123.0 (C20/C22), 123.0 (C20/C22), 119.5 (C13), 109.3 (C24), 102.6 (C7), 97.3 (C2), 82.5 (C5, C6), 81.4 (C3, C4), 50.0 (C26), 47.8 (C14), 38.0 (C17), 30.7 (C8), 28.0 (C16), 27.0 (C15), 22.2 (C9, C10), 18.5 (C1).

Elem. anal. Calc. (%) for C_32_H_35_Cl_4_N_5_ORu: C 51.35, H 4.71 and N 9.36. Found: C 51.16, H 4.86, and N 8.99.

ESI-MS: *m*/*z* 714 [M − Cl]^+^.

**(η^6^-*p*-cymene){*N*-(6-(1*H*-imidazol-1-yl)hexyl)-1-(2,4-dichlorobenzyl)-1*H*-indazole-3-carboxamide}ruthenium(II)-*N* dichloride (10)**


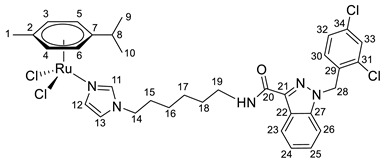


*N*-(6-(1*H*-imidazol-1-yl)hexyl)-1-(2,4-dichlorobenzyl)-1*H*-indazole-3-carboxamide **4** (80 mg, 0.17 mmol) in 5 mL CH_2_Cl_2_ was added to the dimer (η^6^-*p*-cymene-RuCl_2_)_2_ solution (52 mg, 0.085 mmol) in 5 mL CH_2_Cl_2_. The reaction mixture was stirred for 10 h. The solution was evaporated to 1 mL, and 10 mL of ether and 15 mL of hexane were added. The resulting orange precipitate was filtered off, washed with hexane, and dried in a vacuum. Yield 90 mg (68%), T_dec_. = 82–83 °C.

^1^H NMR (400.13 MHz, CDCl_3_) δ: 8.40 (d, 1H, J = 8.1 Hz, H23), 7.88 (s, 1H, H11), 7.47–7.23 (m, 5H, H24–26, H33, H12), 7.14–6.97 (m, 2H, H32, NH), 6.86 (s, 1H, H13), 6.63 (d, 1H, J = 8.3 Hz, H30), 5.65 (s, 2H, H28), 5.42 (d, 2H, J = 5.6 Hz, H5, H6), 5.23 (d, 2H, J = 5.6 Hz, H3, H4), 3.85 (t, 2H, J = 7.1 Hz, H14), 3.52–3.40 (m, 2H, H19), 3.02–2.87 (m, 1H, H8), 2.16 (s, 3H, H1), 1.82–1.70 (m, 2H, H18), 1.69–1.55 (m, 2H, H15), 1.47–1.17 (m, 10H, H9, H10, H16, H17).

^13^C{^1^H} NMR (100.61 MHz, CDCl_3_) δ: 162.5 (C20), 141.2 (C21), 139.7 (C11), 138.5 (C27), 134.5 (C29), 133.2 (C31/C34), 132.4 (C31/C34), 132.1 (C33), 129.5 (C30), 129.4 (C12), 127.6 (C32), 127.4 (C25), 123.1 (C23), 123.0 (C22, C24), 119.4 (C13), 109.2 (C26), 102.5 (C7), 97.3 (C2), 82.6 (C5, C6), 81.4 (C3, C4), 50.1 (C28), 48.2 (C14), 38.7 (C19), 30.7 (C8), 30.4 (C18), 29.6(C15), 26.3 (C16/C17), 26.1 (C16/C17), 22.2 (C9, C10), 18.5 (C1).

Elem. anal. Calc. (%) for C_34_H_39_Cl_4_N_5_ORu: C 52.58, H 5.06, and N 9.01. Found: C 52.29, H 5.16, and N 8.57.

ESI-MS: *m*/*z* 742 [M − Cl]^+^.

**(η^6^-*p*-cymene){*N*-(8-(1*H*-imidazol-1-yl)octyl)-1-(2,4-dichlorobenzyl)-1*H*-indazole-3-carboxamide}ruthenium(II)-*N* dichloride (11)**


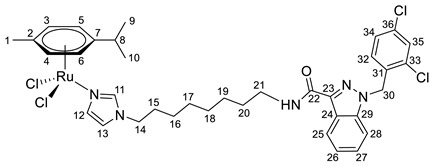


*N*-(8-(1*H*-imidazol-1-yl)octyl)-1-(2,4-dichlorobenzyl)-1*H*-indazole-3-carboxamide **5** (100 mg, 0.2 mmol) in 5 mL CH_2_Cl_2_ was added to the dimer (η^6^-*p*-cymene-RuCl_2_)_2_ solution (61 mg, 0.1 mmol) in 5 mL of CH_2_Cl_2_. The reaction mixture was stirred for 10 h. The solution was evaporated to 1 mL, and 10 mL of ether and 15 mL of hexane were added. The resulting orange precipitate was filtered off, washed with hexane, and dried in a vacuum. Yield 133 mg (82%), T_dec_. = 63–65 °C.

^1^H NMR (400.13 MHz, CDCl_3_) δ: 8.44 (d, 1H, J = 8.1 Hz, H25), 7.91 (s, 1H, H11), 7.49–7.29 (m, 5H, H26–28, H35, H12), 7.13 (dd, 1H, J = 8.3, 2.0 Hz, H34), 7.02 (t, 1H, J = 6.4 Hz, NH), 6.88 (s, 1H, H13), 6.64 (d, 1H, J = 8.3 Hz, H32), 5.69 (s, 2H, H30), 5.45 (d, 2H, J = 5.9 Hz, H5, H6), 5.25 (d, 2H, J = 5.9 Hz, H3, H4), 3.88 (t, 2H, J = 7.4 Hz, H14), 3.50 (q, 2H, J = 6.8 Hz, H21), 3.03–2.93 (m, 1H, H8), 2.19 (s, 3H, H1), 1.82–1.71 (m, 2H, H20), 1.70–1.61 (m, 2H, H15), 1.46–1.24 (m, 14H, H9, H10, H16, H17, H18, H19).

^13^C{^1^H} NMR (100.61 MHz, CDCl_3_) δ: 162.5 (C22), 141.3 (C23), 139.8 (C11), 138.7 (C29), 134.6 (C31), 133.3 (C33/C36), 132.6 (C33/C36), 132.3 (C35), 129.6 (C32), 129.4 (C12), 127.6 (C34), 127.6 (C27), 123.3 (C25), 123.2 (C24/C26), 123.1 (C24/C26), 119.5 (C13), 109.3 (C28), 102.6 (C7), 97.5 (C2), 82.8 (C5, C6), 81.5 (C3, C4), 50.2 (C30), 48.4 (C14), 39.1 (C21), 30.8 (C8), 30.6 (C20), 29.9(C15), 29.2 (C16–20), 29.0 (C16–20), 26.9 (C16–20), 26.5 (C16–20), 22.4 (C9, C10), 18.7 (C1).

Elem. anal. Calc. (%) for C_36_H_43_Cl_4_N_5_ORu*0.1CH_2_Cl_2_: C 53.32, H 5.35, and N 8.61. Found: C 53.04, H 5.45, and N 8.32.

ESI-MS: *m*/*z* 770 [M − Cl]^+^.

**(η^6^-*p*-cymene){*N*-(3-(1*H*-imidazol-1-yl)propyl)-1-(2,4-dichlorobenzyl)-1*H*-indazole-3-carboxamide}ruthenium(II)-*N* oxalate (12)**


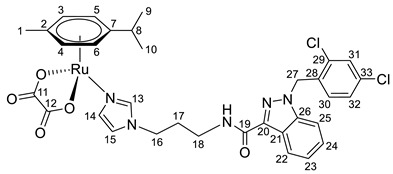


Silver oxalate Ag_2_C_2_O_4_ (67 mg, 0.22 mmol) was added to the dimer (η^6^-*p*-cymene-RuCl_2_)_2_ solution (69 mg; 0.11 mmol) in 40.0 mL of H_2_O. The reaction mixture was stirred for 12 h. Precipitated AgCl was filtered off, and the solvent was evaporated under a vacuum. The resulting ruthenium complex was dissolved in 18.0 mL of MeOH, and a solution of compound **2** (100 mg, 0.22 mmol) in 2.0 mL of MeOH was added. The reaction mixture was stirred for 8 h, the solvent was evaporated under a vacuum, and the product was precipitated with hexane and filtered. The resulting orange precipitate was dried in a vacuum. Yield 82 mg (76%), T_dec_. = 67–70 °C.

^1^H NMR (400.13 MHz, CDCl_3_) δ: 8.39 (d, 1H, J = 8.1 Hz, H22), 7.45–7.30 (m, 5H, H23–25, H31, H14), 7.17 (m, 1H, NH), 7.01 (s, 1H, H13), 6.95 (m, 1H, H32), 6.82 (d, 1H, J = 8.4 Hz, H30), 6.68 (s, 1H, H15), 5.70 (s, 2H, H27), 5.52 (d, 2H, J = 6.0 Hz, H5, H6), 5.35 (d, 2H, J = 6.0 Hz, H3, H4), 4.03 (t, 2H, J = 6.7 Hz, H16), 3.45 (m, 2H, H18), 2.82 (m, 1H, H8), 2.17 (s, 3H, H1), 1.28 (m, 8H, H9, H10, H17).

^13^C{^1^H} NMR (100.61 MHz, CDCl_3_) δ: 165.9 (C11, C12), 163.0 (C19), 141.1 (C20), 139.3 (C13), 138.0 (C26), 134.5 (C28), 133.2 (C29/C33), 132.3 (C29/C33), 130.0 (C31), 129.7 (C30), 129.4 (C14), 127.8 (C32), 127.4 (C24), 123.0 (C22), 123.0 (C21, C23), 120.5 (C15), 109.5 (C25), 100.9 (C7), 96.9 (C2), 82.3 (C5, C6), 80.0 (C3, C4), 50.0 (C27), 45.4 (C16), 35.4 (C18), 30.9 (C8), 27.7 (C17), 22.5 (C9, C10), 17.9 (C1).

Elem. anal. Calc. (%) for C_33_H_33_Cl_2_N_5_O_5_Ru: C 52.73, H 4.43, and N 9.32. Found: C 52.70, H 4.70, and N 8.83.

ESI-MS: *m*/*z* 774 [M + Na]^+^.

**(η^6^-*p*-cymene){*N*-(6-(1*H*-imidazol-1-yl)hexyl)-1-(2,4-dichlorobenzyl)-1*H*-indazole-3-carboxamide}ruthenium(II)-*N* oxalate (13)**


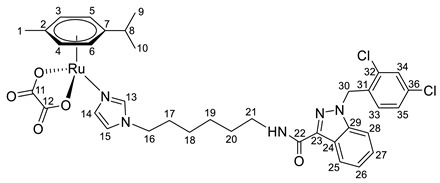


Silver oxalate Ag_2_C_2_O_4_ (67 mg, 0.22 mmol) was added to the dimer (η^6^-*p*-cymene-RuCl_2_)_2_ solution (69 mg; 0.11 mmol) in 40.0 mL H_2_O. The reaction mixture was stirred for 12 h. Precipitated AgCl was filtered off, and the solvent was evaporated under a vacuum. The resulting ruthenium complex was dissolved in 18.0 mL of MeOH, and a solution of compound **4** (100 mg, 0.22 mmol) in 2.0 mL of MeOH was added. The reaction mixture was stirred for 8 h, the solvent was evaporated under a vacuum, and the product was precipitated with hexane and filtered. The resulting orange precipitate was dried in a vacuum. Yield 78 mg (72%).

^1^H NMR (400.13 MHz, CDCl_3_) δ: 8.29 (d, 1H, J = 8.5 Hz, H25), 7.48–7.29 (m, 5H, H26–28, H34, H14), 7.12 (m, 2H, H35, NH), 7.05 (s, 1H, H13), 6.90 (d, 1H, J = 8.4 Hz, H33), 6.68 (s, 1H, H15), 5.67 (s, 2H, H30), 5.48 (d, 2H, J = 5.8 Hz, H5, H6), 5.26 (d, 2H, J = 5.6 Hz, H3, H4), 3.89 (t, 2H, J = 7.7 Hz, H16), 3.52–3.45 (m, 2H, H21), 2.80–2.79 (m, 1H, H8), 2.17 (s, 3H, H1), 1.66–1.61 (m, 2H, H17), 1.42–1.40 (m, 2H, H20), 1.29–1.28 (m, 10H, H9, H10, H18, H19).

^13^C{^1^H} NMR (100.61 MHz, CDCl_3_) δ: 165.7 (C11, C12), 162.5 (C22), 141.1 (C23), 138.4 (C13), 138.1 (C29), 134.5 (C31), 133.2 (C32/C36), 132.4 (C32/C36), 130.6 (C34), 129.6 (C33), 129.4 (C14), 127.6 (C35), 127.4 (C27), 123.0 (C25), 122.6 (C24, C26), 120.5 (C15), 109.3 (C28), 100.9 (C7), 97.0 (C2), 82.3 (C5, C6), 79.8 (C3, C4), 50.0 (C30), 48.3 (C16), 38.7 (C21), 30.9 (C8), 30.4 (C20), 29.5 (C17), 26.2 (C18/C19), 25.9 (C18/C19), 22.5 (C9, C10), 18.0 (C1).

Elem. anal. Calc. (%) for C_36_H_39_Cl_2_N_5_O_5_Ru: C 54.48, H 4.95, and N 8.82. Found: C 54.02, H 4.89, and N 8.56.

ESI-MS: *m*/*z* 816 [M + Na]^+^.

**(η^6^-*p*-cymene){*N*-(12-(1*H*-imidazol-1-yl)dodecyl)-1-(2,4-dichlorobenzyl)-1*H*-indazole-3-carboxamide}ruthenium(II)-*N* oxalate (14)**


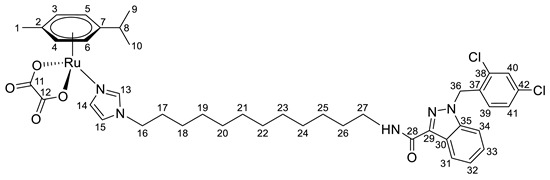


Silver oxalate Ag_2_C_2_O_4_ (67 mg, 0.22 mmol) was added to the dimer (η^6^-*p*-cymene-RuCl_2_)_2_ solution (69 mg; 0.11 mmol) in 40.0 mL of H_2_O. The reaction mixture was stirred for 12 h. Precipitated AgCl was filtered off, and the solvent was evaporated under a vacuum. The resulting ruthenium complex was dissolved in 18.0 mL of MeOH, and a solution of compound **6** (122 mg, 0.22 mmol) in 2.0 mL of MeOH was added. The reaction mixture was stirred for 8 h, the solvent was evaporated under a vacuum, and the product was precipitated with hexane and filtered. The resulting orange precipitate was dried in a vacuum. Yield 98 mg (65%), T_melt_. = 84–86 °C.

^1^H NMR (400.13 MHz, CDCl_3_) δ: 8.42 (d, 1H, J = 8.1 Hz, H32), 7.44–7.27 (m, 5H, H14, H33–35, H41), 7.13–7.10 (m, 2H, H42, NH), 7.05 (s, 1H, H15), 6.91 (d, 1H, J = 8.3 Hz, H40), 6.43 (s, 1H, H16), 5.66 (s, 2H, H37), 5.47 (d, 2H, J = 5.7 Hz, H5, H6), 5.26 (d, 2H, J = 5.9 Hz, H3, H4), 3.86 (t, 2H, J = 7.3 Hz, H17), 3.48 (q, 2H, J = 6.9 Hz, H28), 2.83–2.77 (m, 1H, H8), 2.16 (s, 3H, H1), 1.78–1.63 (m, 4H, H18, H27), 1.45–1.23 (m, 22H, H19–26, H9, H10).

^13^C{^1^H} NMR (100.61 MHz, CDCl_3_) δ: 165.4 (C11, C12), 162.3 (C28), 141.1 (C13), 138.5 (C29), 137.9 (C35), 134.4 (C37), 133.1 (C42), 132.4 (C38), 130.5 (C14), 129.4 (C39), 129.3 (C33), 127.5 (C41), 127.4 (C40), 123.1 (C31), 123.1 (C30), 123.0 (C32), 120.4 (C15), 109.3 (C34), 100.7 (C7), 96.9 (C2), 82.3 (C5, C6), 79.8 (C3, C4), 50.0 (C36), 48.4 (C16), 39.1 (C27), 30.9 (C8), 30.6 (C17), 29.8 (C26), 28.8 (C18–25), 28.7 (C18–25), 28.6 (C18–25), 28.6 (C18–25), 28.2 (C18–25), 27.2 (C18–25), 26.6 (C18–25), 22.5 (C9, C10), 18.0 (C1).

Elem. anal. Calc. (%) for C_38_H_43_Cl_2_N_5_O_5_Ru*0.7CH_3_OH: C 56.97, H 6.02, and N 7.78. Found: C 56.53, H 5.65, and N 7.94.

ESI-MS: *m*/*z* 900 [M + Na]^+^.

**(η^6^-*p*-cymene){*N*-(3-(1*H*-imidazol-1-yl)propyl)-1-(2,4-dichlorobenzyl)-1*H*-indazole-3-carboxamide}ruthenium(II)-*N* malonate (15)**


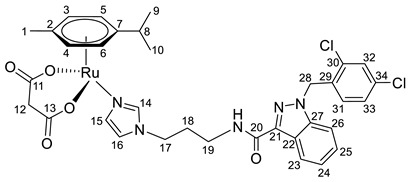


Silver malonate Ag_2_C_3_H_2_O_4_ (70 mg, 0.2195 mmol) was added to the dimer (η^6^-*p*-cymene-RuCl_2_)_2_ solution (67 mg; 0.1097 mmol) in 40.0 mL of H_2_O. The reaction mixture was stirred for 12 h. Precipitated AgCl was filtered off, and the solvent was evaporated under a vacuum. The resulting ruthenium complex was dissolved in 18.0 mL of MeOH, and a solution of compound **2** (94 mg, 0.2195 mmol) in 2.0 mL of MeOH was added. The reaction mixture was stirred for 8 h, the solvent was evaporated under a vacuum, and the product was isolated by column chromatography on silica gel (eluent: EtOAc:MeOH:CH_2_Cl_2_ 3:3:1, Rf = 0.5). The resulting orange precipitate was dried in a vacuum. Yield 97 mg (58%), T_melt_. = 115–117 °C.

^1^H NMR (400.13 MHz, CDCl_3_) δ: 8.37 (d, 1H, J = 8.2 Hz, H23), 7.75 (s, 1H, H14), 7.45–7.27 (m, 4H, H24–26, H32), 7.17–7.13 (m, 3H, H33, H15, NH), 7.02 (s, 1H, H16), 6.74 (d, 1H, J = 8.4 Hz, H31), 5.69 (s, 2H, H28), 5.52 (d, 2H, J = 6.0 Hz, H5, H6), 5.33 (d, 2H, J = 6.0 Hz, H3, H4), 4.02 (t, 2H, J = 6.8 Hz, H17), 3.46 (q, 2H, J = 6.4 Hz, H19), 3.38 (d, 1H, J = 15.9 Hz, H12), 2.85–2.77 (m, 2H, H12, H8), 2.16 (s, 3H, H1), 2.11–2.04 (m, 2H, H18), 1.27 (d, 6H, J = 6.9 Hz, H9, H10).

^13^C{^1^H} NMR (100.61 MHz, CDCl_3_) δ: 175.1 (C11, C13), 163.0 (C20), 141.2 (C14), 139.1 (C21), 137.9 (C27), 134.6 (C29), 133.3 (C30/C34), 132.2 (C30/C34), 130.3 (C15), 129.7 (C31), 129.5 (C25), 127.8 (C33), 127.6 (C32), 123.2 (C23), 123.0 (C22/C24), 122.8 (C22/C24), 120.0 (C16), 109.5 (C26), 101.4 (C7), 97.1 (C2), 82.3 (C5, C6), 80.5 (C3, C4), 50.2 (C28), 46.7 (C12), 45.5 (C17), 35.3 (C19), 31.1 (C18), 30.7 (C8), 22.4 (C9, C10), 18.0 (C1).

Elem. anal. Calc. (%) for C_34_H_35_Cl_2_N_5_O_5_Ru*0.1CH_2_Cl_2_: C 52.91, H 4.58, and N 9.05. Found: C 52.68, H 4.50, and N 8.97.

ESI-MS: *m*/*z* 766 [M + H]^+^, *m*/*z* 788 [M + Na]^+^.

**(η^6^-*p*-cymene){*N*-(6-(1*H*-imidazol-1-yl)hexyl)-1-(2,4-dichlorobenzyl)-1*H*-indazole-3-carboxamide}ruthenium(II)-*N* malonate (16)**


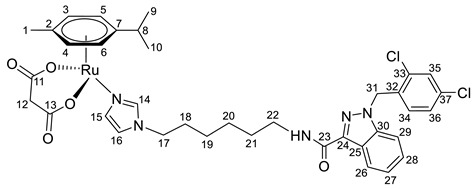


Silver malonate Ag_2_C_3_H_2_O_4_ (86 mg, 0.2722 mmol) was added to the dimer (η^6^-*p*-cymene-RuCl_2_)_2_ solution (83 mg; 0.1361 mmol) in 50.0 mL of H_2_O. The reaction mixture was stirred for 12 h. Precipitated AgCl was filtered off, and the solvent was evaporated under a vacuum. The resulting ruthenium complex was dissolved in 22.0 mL of MeOH, and a solution of compound **4** (128 mg, 0.2722 mmol) in 3.0 mL of MeOH was added. The reaction mixture was stirred for 8 h, the solvent was evaporated under a vacuum, and the product was isolated by column chromatography on silica gel (eluent: EtOAc:MeOH:CH_2_Cl_2_ 3:3:1, Rf = 0.5). The resulting orange precipitate was dried in a vacuum. Yield 138 mg (62%), T_melt_. = 110–113 °C.

^1^H NMR (400.13 MHz, CDCl_3_) δ: 8.40 (d, 1H, J = 8.2 Hz, H26), 7.56 (s, 1H, H14), 7.45–7.27 (m, 4H, H27–29, H35), 7.20 (s, 1H, H15), 7.12–7.07 (m, 2H, H36, NH), 6.93 (s, 1H, H16), 6.65 (d, 1H, J = 8.4 Hz, H34), 5.66 (s, 2H, H31), 5.47 (d, 2H, J = 5.9 Hz, H5, H6), 5.26 (d, 2H, J = 5.9 Hz, H3, H4), 3.91 (t, 2H, J = 7.2 Hz, H17), 3.47 (q, 2H, J = 6.8 Hz, H22), 3.36 (d, 1H, J = 16.1 Hz, H12), 2.83–2.77 (m, 2H, H8), 2.75 (d, 1H, J = 16.1 Hz, H12), 2.14 (s, 3H, H1), 1.81–1.74 (m, 2H, H18), 1.67–1.61 (m, 2H, H21), 1.45–1.39 (m, 2H, H19), 1.35–1.29 (m, 2H, H20), 1.26 (d, 6H, J = 6.9 Hz, H9, H10).

^13^C{^1^H} NMR (100.61 MHz, CDCl_3_) δ: 175.0 (C11, C13), 162.5 (C23), 141.2 (C14), 138.5 (C24), 138.0 (C30), 134.5 (C32), 133.2 (C37), 132.4 (C33), 130.8 (C15), 129.5 (C34), 129.4 (C28), 127.6 (C36), 127.4 (C35), 123.1 (C26), 123.1 (C25), 123.0 (C27), 120.2 (C16), 109.3 (C29), 101.4 (C7), 97.2 (C2), 82.3 (C5, C6), 80.4 (C3, C4), 50.1 (C31), 48.4 (C17), 46.5 (C12), 38.7 (C22), 30.7 (C8), 30.5 (C18), 29.6 (C21), 26.2 (C19), 26.0 (C20), 22.4 (C9, C10), 18.0 (C1).

Elem. anal. Calc. (%) for C_37_H_41_Cl_2_N_5_O_5_Ru: C 55.02, H 5.12, and N 8.67. Found: C 54.97, H 4.98, and N 8.58.

ESI-MS: *m*/*z* 810 [M + H]^+^, 830 [M + Na]^+^.

**(η^6^-*p*-cymene){*N*-(12-(1*H*-imidazol-1-yl)dodecyl)-1-(2,4-dichlorobenzyl)-1*H*-indazole-3-carboxamide}ruthenium(II)-*N* malonate (17)**


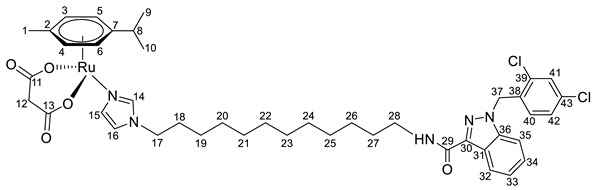


Silver malonate Ag_2_C_3_H_2_O_4_ (96 mg, 0.3029 mmol) was added to the dimer (η^6^-*p*-cymene-RuCl_2_)_2_ solution (93 mg; 0.1515 mmol) in 60.0 mL of H_2_O. The reaction mixture was stirred for 12 h. Precipitated AgCl was filtered off, and the solvent was evaporated under a vacuum. The resulting ruthenium complex was dissolved in 27.0 mL of MeOH, and a solution of compound **6** (168 mg, 0.3029 mmol) in 3.0 mL of MeOH was added. The reaction mixture was stirred for 8 h, the solvent was evaporated under a vacuum, and the product was isolated by column chromatography on silica gel (eluent: EtOAc:MeOH:CH_2_Cl_2_ 3:3:1, Rf = 0.5). The resulting orange precipitate was dried in a vacuum. Yield 171 mg (63%), T_melt_. = 90–94 °C.

^1^H NMR (400.13 MHz, CDCl_3_) δ: 8.42 (d, 1H, J = 8.1 Hz, H32), 7.53 (s, 1H, H14.), 7.45–7.27 (m, 4H, H33–35, H41), 7.21 (s, 1H, H15), 7.10 (dd, 1H, J = 8.4, 1.9 Hz, H42), 7.00 (t, 1H, J = 5.7 Hz, NH), 6.93 (s, 1H, H16), 6.62 (d, 1H, J = 8.3 Hz, H40), 5.66 (s, 2H, H37), 5.46 (d, 2H, J = 5.7 Hz, H5, H6), 5.25 (d, 2H, J = 5.9 Hz, H3, H4), 3.89 (t, 2H, J = 7.3 Hz, H17), 3.48 (q, 2H, J = 6.9 Hz, H28), 3.36 (d, 1H, J = 16.1 Hz, H12), 2.83–2.77 (m, 1H, H8), 2.75 (d, 1H, J = 16.1 Hz, H12), 2.14 (s, 3H, H1), 1.78–1.71 (m, 2H, H18), 1.68–1.61 (m, 2H, H27), 1.43–1.22 (m, 22H, H19–26, H9, H10).

^13^C{^1^H} NMR (100.61 MHz, CDCl_3_) δ: 174.0 (C11, C13), 161.4 (C29), 140.2 (C14), 137.6 (C30), 136.9 (C36), 133.5 (C38), 132.2 (C43), 131.5 (C39), 129.8 (C15), 128.5 (C40), 128.3 (C34), 126.6 (C42), 126.4 (C41), 122.2 (C32), 122.1 (C31), 122.0 (C33), 119.2 (C16), 108.2 (C35), 100.3 (C7), 96.2 (C2), 81.3 (C5, C6), 79.3 (C3, C4), 49.0 (C37), 47.5 (C17), 45.5 (C12), 38.1 (C28), 29.7 (C8), 29.6 (C18), 28.8 (C27), 28.5 (C19–26), 28.4 (C19–26), 28.3 (C19–26), 28.3 (C19–26), 27.9 (C19–26), 26.0 (C19–26), 25.4 (C19–26), 21.4 (C9, C10), 17.0 (C1).

Elem. anal. Calc. (%) for C_43_H_53_Cl_2_N_5_O_5_Ru: C 57.91, H 5.99, and N 7.85. Found: C 57.63, H 5.90, and N 7.77.

ESI-MS: *m*/*z* 892 [M + H]^+^, *m*/*z* 914 [M + Na]^+^.

**(η^6^-*p*-cymene)bis-{*N*-(3-(1*H*-imidazol-1-yl)propyl)-1-(2,4-dichlorobenzyl)-1*H*-indazole-3-carboxamide}ruthenium(II)-*N* chloride (18)**


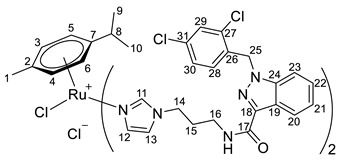


*N*-(3-(1*H*-imidazol-1-yl)propyl)-1-(2,4-dichlorobenzyl)-1*H*-indazole-3-carboxamide **2** (123 mg, 0.2872 mmol) in 2.0 mL of CH_2_Cl_2_ was added to the dimer (η^6^-*p*-cymene-RuCl_2_)_2_ solution (44 mg, 0.0718 mmol) in 23.0 mL of CH_2_Cl_2_. The reaction mixture was stirred for 5 h, the solvent was evaporated under a vacuum, and the product was isolated by column chromatography on silica gel (eluent: CH_2_Cl_2_:MeOH 10:1, R_f_ = 0.5). The resulting orange precipitate was dried in a vacuum. Yield 132 mg (79%), T_melt_. = 100–103 °C.

^1^H NMR (400.13 MHz, CDCl_3_) δ: 9.35 (s, 2H, H11), 8.28 (d, 2H, J = 8.2 Hz, H20), 7.73 (t, 2H, J = 6.0 Hz, NH), 7.44 (s, 2H, H12), 7.34–7.17 (m, 8H, H21–23, H29), 7.04 (dd, 2H, J = 8.3, 1.9 Hz, H30), 6.82 (s, 2H, H13), 6.73 (d, 2H, J = 8.4 Hz, H28), 5.77 (s, 2H, H5, H6), 5.57 (s, 2H, H3, H4), 4.17–4.04 (m, 4H, H14), 3.44–3.31 (m, 4H, H16), 2.42–2.34 (m, 1H, H8), 2.18–2.09 (m, 4H, H15), 1.74 (s, 3H, H1), 1.07 (d, 6H, J = 6.9 Hz, H9, H10).

^13^C{^1^H} NMR (100.61 MHz, CDCl_3_) δ: 162.0 (C17), 140.9 (C11), 140.0 (C18), 137.2 (C24), 133.4 (C26), 132.1 (C27/C31), 131.3 (C27/C31), 129.2 (C12), 128.9 (C28), 128.3 (C22), 126.7 (C30), 126.3 (C29), 122.0 (C20), 122.0 (C19/C21), 121.8 (C19/C21), 118.5 (C13), 108.3 (C23), 102.1 (C7), 99.6 (C2), 85.2 (C5, C6), 81.0 (C3, C4), 49.0 (C25), 44.8 (C14), 34.8 (C16), 29.9 (C15), 29.9 (C8), 21.3 (C9, C10), 16.9 (C1).

Elem. anal. Calc. (%) for C_52_H_52_Cl_6_N_10_O_2_Ru*0.7CH_2_Cl_2_: C 51.79, H 4.40, and N 11.46. Found: C 51.85, H 4.45, and N 11.65.

ESI-MS: *m*/*z* 1127 [M − Cl]^+^.

**(η^6^-*p*-cymene)bis-{*N*-(12-(1*H*-imidazol-1-yl)dodecyl)-1-(2,4-dichlorobenzyl)-1*H*-indazole-3-carboxamide}ruthenium(II)-*N* chloride (19)**


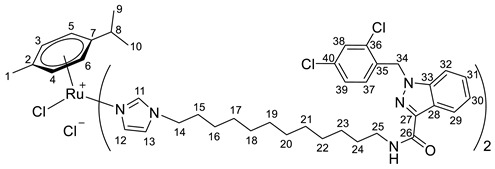


*N*-(12-(1*H*-imidazol-1-yl)dodecyl)-1-(2,4-dichlorobenzyl)-1*H*-indazole-3-carboxamide **6** (155 mg, 0.2795 mmol) in 2.0 mL of CH_2_Cl_2_ was added to the dimer (η^6^-*p*-cymene-RuCl_2_)_2_ solution (43 mg, 0.0699 mmol) in 23.0 mL CH_2_Cl_2_. The reaction mixture was stirred for 5 h, the solvent was evaporated under a vacuum, and the product was isolated by column chromatography on silica gel (eluent: CH_2_Cl_2_:MeOH 10:1, R_f_ = 0.5). The resulting orange precipitate was dried in a vacuum. Yield 137 mg (69%), T_melt_. = 70–73 °C.

^1^H NMR (400.13 MHz, CDCl_3_) δ: 9.13 (s, 2H, H11), 8.42 (d, 2H, J = 8.1 Hz, H29), 7.63 (s, 2H, H12), 7.46–7.27 (m, 8H, H30–32, H38), 7.09 (dd, 2H, J = 8.4, 1.9 Hz, H39), 7.01 (t, 2H, J = 5.5 Hz, NH), 6.79 (s, 2H, H13), 6.60 (d, 2H, J = 8.4 Hz, H37), 5.88 (d, 2H, J = 5.8 Hz, H5, H6), 5.84 (d, 2H, J = 5.8 Hz, H3, H4), 5.65 (s, 4H, H34), 4.03–3.94 (m, 4H, H14), 3.47 (q, 4H, J = 7.0 Hz, H25), 2.40–2.32 (m, 1H, H8), 1.78–1.60 (m, 11H, H15, H24, H1), 1.44–1.16 (m, 32H, H16–23), 1.12 (d, 6H, J = 6.9 Hz, H9, H10).

^13^C{^1^H} NMR (100.61 MHz, CDCl_3_) δ: 161.3 (C26), 140.4 (C11), 140.1 (C27), 137.6 (C33), 133.4 (C35), 132.1 (C36/C40), 131.4 (C36/C40), 129.6 (C12), 128.4 (C37), 128.2 (C31), 126.6 (C39), 126.4 (C38), 122.2 (C29), 122.0 (C28), 121.9 (C30), 118.2 (C13), 108.1 (C32), 102.3 (C7), 99.3 (C2), 84.9 (C5, C6), 81.4 (C3, C4), 49.0 (C34), 47.2 (C14), 38.1 (C25), 29.8 (C8), 29.7 (C15), 28.8 (C24), 28.5 (C16–23), 28.5 (C16–23), 28.5 (C16–23), 28.5 (C16–23), 28.3 (C16–23), 28.0 (C16–23), 26.0 (C16–23), 25.3 (C16–23), 21.2 (C9, C10), 16.8 (C1).

Elem. anal. Calc. (%) for C_70_H_88_Cl_6_N_10_O_2_Ru*0.7CH_2_Cl_2_: C 57.58, H 6.11, and N 9.50. Found: C 57.90, H 5.60, and N 9.75.

ESI-MS: *m*/*z* 1379 [M − Cl]^+^.

**(η^6^-*p*-cymene){*N*-(3-(1*H*-imidazol-1-yl)propyl)acetamide}ruthenium(II)-*N* oxalate (21)**


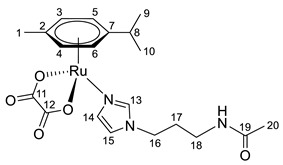


Silver oxalate Ag_2_C_2_O_4_ (70 mg, 0.2282 mmol) was added to the dimer (η^6^-*p*-cymene-RuCl_2_)_2_ solution (70 mg; 0.1141 mmol) in 40.0 mL of H_2_O. The reaction mixture was stirred for 12 h. Precipitated AgCl was filtered off, and the solvent was evaporated under a vacuum. The resulting ruthenium complex was dissolved in 18.0 mL of MeOH, and a solution of compound **20** [51] (38 mg, 0.2282 mmol) in 2.0 mL of MeOH was added. The reaction mixture was stirred for 8 h, the solvent was evaporated under a vacuum, and the product was isolated by column chromatography on silica gel (eluent: MeOH:CH_2_Cl_2_ 1:9, Rf = 0.5). The resulting orange precipitate was dried in a vacuum. Yield 85 mg (76%).

^1^H NMR (400.13 MHz, CDCl_3_) δ: 7.76 (t, 1H, J = 5.4 Hz, NH), 7.66 (s, 1H, H13), 6.93 (s, 1H, H14), 6.82 (s, 1H, H15), 5.56 (d, 2H, J = 6.1 Hz, H5, H6), 5.39 (d, 2H, J = 6.0 Hz, H3, H4), 3.76 (t, 2H, J = 6.9 Hz, H16), 3.03 (q, 2H, J = 5.8 Hz, H18), 2.83–2.75 (m, 1H, H8), 2.16 (s, 3H, H1), 1.99 (s, 3H, H20), 1.78–1.70 (m, 2H, H17), 1.28 (d, 6H, J = 6.9 Hz, H9, H10).

ESI-MS: *m*/*z* 526 [M + Cl]^−^, 492 [M + H]^+^, 514 [M + Na]^+^.

### 2.2. Log P Determination

Log P values of the new compounds were determined by the HPLC method [42,43] using a Phenomenex Kinetex 5 μ XB-C18 100 Å column 150 × 4.6 mm using two mobile phases: phase A was 20 mM MOPS, 0.15% decylamine, pH = 7.4; phase B was 0.25% 1-octanol in methanol. Briefly, samples dissolved in methanol with uracil as an internal standard were injected into the column and eluted with mobile phase B between 70%, 80%, and 90%. The log P values were calculated as previously described [43] using benzaldehyde, methyl benzoate, ethoxybenzene, naphthalene, and 1-chloronaphthalene as standards. These experiments were repeated three times for each of the compounds.

### 2.3. Cell Death Studies

The antiproliferative activity was studied by MTT assays as published previously [45]. For the flow cytometry studies, cells were plated into 6-well plates (Eppendorf, Germany; HCT-116 cells, 4 × 10^5^ cells in 2 mL of DMEM) and incubated for 24 h. Solutions of complexes in DMSO were prepared immediately prior to the day of the experiments. A Cisplatin solution was prepared in DMEM without the addition of DMSO. Cells were treated with either 20 µM of cisplatin, 25 µM of **12**, 25 µM of **14**, 20 µM of **18**, or 20 µM of **19**. Concentrations corresponded to twofold IC_50_ values based on MTT assays. Cells were incubated for 24, 48 and 72 h, pooled, washed with PBS, and resuspended in DMEM. Aliquots of cells were processed as recommended in the Muse Annexin V&Dead Cell Kit or Muse Caspase-3/7 Kit (Luminex). Measurements were carried out on a Muse Cell Analyser, Luminex corp., Austin, TX, USA according to the manufacturer protocol.

### 2.4. TrxR1 Assay

The activity of rat TrxR1 in the presence of target compounds was determined in vitro using hepatocyte homogenate as we described previously [49].

## 3. Results and Discussion

### Synthesis and Characterization

**Scheme 1:**
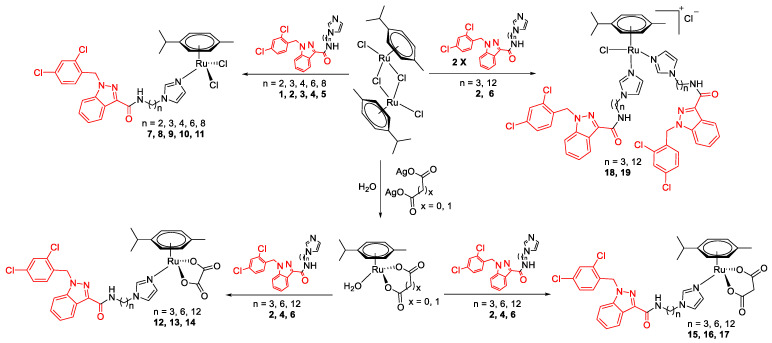
Synthesis of Ru(II) complexes.

Previously, we have reported the synthetic route and antiproliferative activity data for the lonidamine-modified imidazole ligands **1**–**6** [47,49] and utilized them for the preparation of various Ru(III) and Ru(II) compounds, including complex **8** [47]. In this work, new complexes **7**, **9**–**11** were obtained by coordination of imidazole ligands **1**, **3**–**5** with the ruthenium dimer ((η^6^-*p*-cymene)RuCl_2_)_2_ in CH_2_Cl_2_ in the ratio 2:1 (Scheme 1).

Complexes **12**–**17** with the oxalate or malonate moiety were prepared in two steps procedure: first the formation in situ ruthenium aqua complexes from the ruthenium dimer with silver oxalate or malonate, correspondingly, were carried out and later, coordination of aqua complex with ligands **2**, **4**, and **6**. Complexes **18**–**19** were prepared by coordination ligands **2**, **6,** and ((η^6^-*p*-cymene)RuCl_2_)_2_ in the ratio 4:1 (Scheme 1). All obtained complexes, **7**–**19,** were fully characterized with ^1^H and ^13^C{^1^H} NMR spectroscopy, ESI mass-spectrometry, and elemental analysis which have fully confirmed the structure of expected products (see Supplementary Figures S1–S6).

It has been found that Ru(II) organometallic compounds with chloride ligands easily entered into ligand exchange reactions with several solvent molecules, such as water or DMSO [52]. DMSO is widely used in in vitro tests, while the transformation of organometallic compounds in DMSO-containing solutions can hinder the study of biological activity. To overcome the mentioned problem, we have proposed an approach to obtaining analogues resistant to the ligand exchange reactions. This was achieved by replacing the chloride ligands with the dicarboxylic acid moiety or introducing a second imidazole ligand into the coordination sphere.

**Figure 3:**
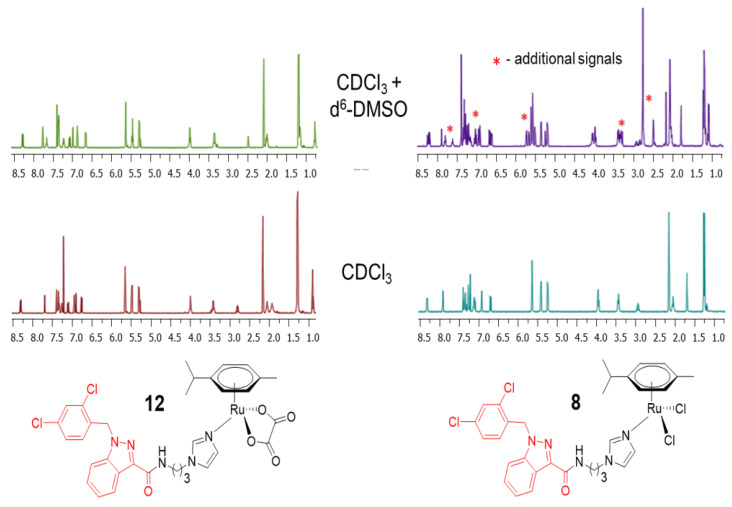
The formation of additional signals in presence of d6-DMSO (signal at δ 2.5 ppm) for **8**, and stability of complex **12** at least for 24 h.

The stability of complexes **7**–**19** in DMSO-containing solutions has been studied by NMR spectroscopy. ^1^H NMR spectra of compounds **7**–**11** bearing two chloride ligands include additional signals corresponding to ligand exchange products when a DMSO-containing solvent is used; whereas, compounds with an oxalate or malonate fragment as well as complexes **18**–**19** with two imidazole ligands do not show any additional signals, hence demonstrating no transformation of the complex in the solution (Figure 3). Complexes **12**–**19** were also found to be stable in pure DMSO.

The lipophilicity of complexes **12**–**14** with oxalate moiety was determined by HPLC (Table 1). For complexes **7**–**11** and **18**, **19** we observed irreversible absorption on the column. Complexes showed high lipophilicity, as was expected, and an increase in Log P values with an increase in linker length.

**Scheme 2:**
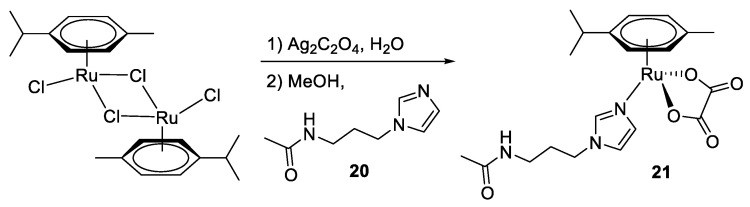
Synthesis of the Ru(II) analogue without lonidamine.

To confirm the key role of lonidamine in the cytotoxicity of the obtained complexes, analogue **21** without lonidamine moiety was obtained (Scheme 2). The complex was synthesized by coordination of ligand **20** to ruthenium aqua complex with an oxalate group obtained in situ.

Cytotoxicity of new Ru(II) complexes was investigated by the MTT assay on human cancer cell lines A549 (non-small cell lung cancer), MCF7 (breast cancer), SW480 (colon carcinoma), and HCT116 (colorectal carcinoma) (Table 2).

**Figure 4:**
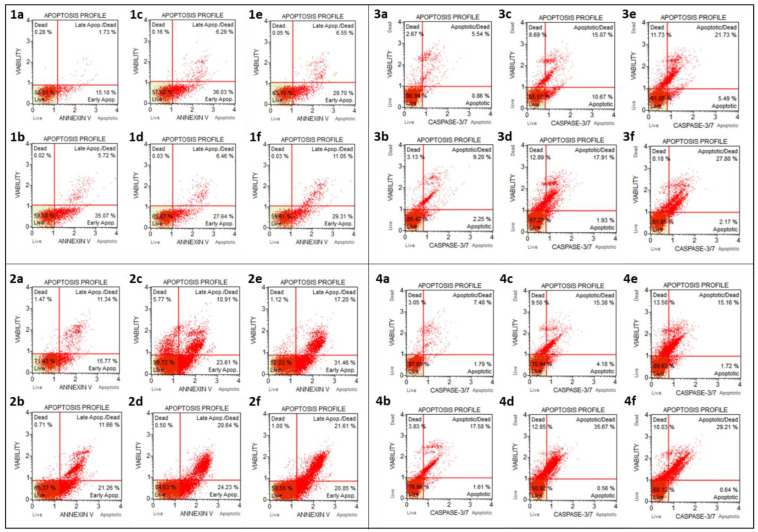
Flow cytometry studies of apoptosis induction on HCT116 cell lines (**1a**–**f** incubation for 48 h, **2a**–**f** incubation for 72 h) and caspase activation (**3a**–**f** incubation for 48 h, **4a**–**f** incubation for 72 h), **1**–**4a**—control, **1**–**4b**—cisplatin (20 µM), **1**–**4c**—complex **12** (25 µM), **1**–**4d**—complex **14** (25 µM), **1**–**4e**—complex **18** (20 µM), **1**–**4f**—complex **19** (20 µM).

Complexes show cytotoxicity in a medium micromolar range exceeding or equal activity of the parent organic drug lonidamine and corresponding ligands **1**–**6** [47,49]. Moreover, in some cases, their activity is higher than the cytotoxicity of cisplatin. Cytotoxicity studies have established that, regardless of the complex stability in the presence of DMSO, cytotoxicity was in a similar range. For complexes **18**–**19**, it was shown that the introduction of the second ligand containing lonidamine into the structure leads to a two-times increase in cytotoxicity in in vitro tests compare to complexes with only one ligand. An increase in the linker length about twice increases activity, however, it significantly raised lipophilicity. Unfortunately, we did not observe any selectivity toward the cancer cells in the experiments with the non-tumorigenic WI38 cell line (IC_50_ 25.05 ± 0.03 for **15**). Moreover, the antiproliferative study confirmed the significance of lonidamine moiety in the compound, as ligand **20** and complex **21** exhibited no activity. For further cell death studies by flow cytometry, complexes **12**, **14**, **18**, and **19** were chosen, and cisplatin was used as a reference drug (Figure 4).

Cytometric studies of apoptosis induction and caspase activation on the HCT116 cell line revealed that Ru(II) organometallic compounds with lonidamine-containing ligands at an early stage (after 24 h of incubation) do not lead to significant apoptosis induction (~15%), and there are no cells with activated caspases (0%). This is probably due to the slow transformation to the active form of the Ru-prodrug. However, after 48 h, and especially after 72 h organometallic derivatives start to show significant apoptosis induction which is accompanied by caspase activation.

Thioredoxin reductases belong to the thioredoxin system and play a crucial role in regulating redox processes, transcription, and protection from reactive oxygen species. TrxR1 is one of the cytosolic isoforms of this enzyme, which is overexpressed in cancer cells, making it a target for developing new anticancer therapies [53]. Due to the presence of the selenocysteine enzyme in the active centre, the majority of known TrxR inhibitors are electrophilic compounds [54], making it necessary to study the inhibitory effect of new compounds on TrxR1 in vitro.

**Figure 5:**
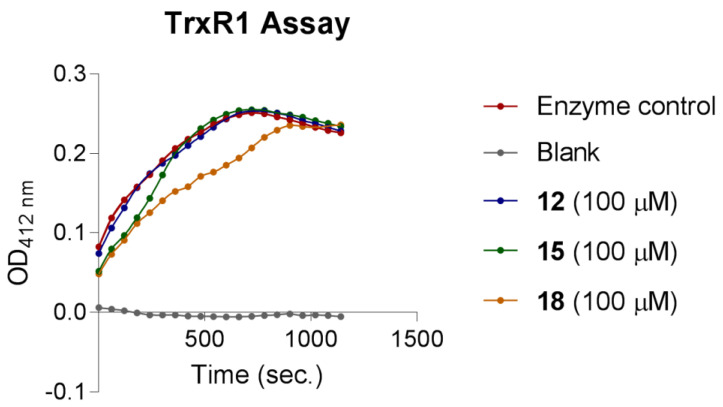
Kinetics of TrxR1 activity in the presence of selected Ru(II) complexes.

To assess whether the engagement of thioredoxin reductase 1 contributes to the cytotoxic action of novel Ru(II) complexes, we evaluated selected compounds as TrxR1 inhibitors in a functional in vitro assay. Complexes **12**, **15**, and **18**, which comprise lonidamine/oxalate, lonidamine/malonate, and bis-lonidamine moieties, respectively, were tested at a final concentration of 100 μM (Figure 5). We have found that these Ru(II) complexes lack significant TrxR1 inhibitory properties regardless of the ligand’s nature, unlike previously reported Ru(III) complexes [40]. Thus, it appears that the ruthenium oxidation state and the presence of the Ru-C bond play a definitive role in the compounds’ mechanism of action.

## 4. Conclusions

The ruthenium organometallic compounds with lonidamine ligand connected by an imidazole linker were prepared. The presence of oxalate, malonate moiety, or second lonidamine ligand leads to high stability in the ligand exchange reaction. These complexes showed good antiproliferative activity and high lipophilicity but also some increase in activity with an increase in the length of the linker. The study on the mechanism of cell death revealed slow induction of apoptosis without activation of caspases. In contrast to previously studied Ru(III) complexes with the same ligand, the TrxR1 is not inhibited by Ru(II) organometallic analogues. The new compounds described herein represent an interesting and promising class of antiproliferative ruthenium complexes that will be studied further, including in vivo evaluation.

## Figures and Tables

**Table 1:** The lipophilicity of complexes.

Compound	12	13	14
Log P	3.64	5.51	8.29

**Table 2:** Antiproliferative activity against human tumour cells.

Compound	Linker, n	IC_50_, μM			
		A549	MCF7	SW480	HCT116
cisplatin		9 ± 1	13 ± 1	22 ± 1	12 ± 1
Lonidamin [46]		>90	30 ± 10	>90	nd
**7**	2	39 ± 2	41 ± 2	34 ± 4	nd
**8**	3	37 ± 4	18 ± 3	30 ± 7	nd
**9**	4	29 ± 6	20 ± 3	25.0 ± 0.3	nd
**10**	6	55 ± 3	48 ± 1	41 ± 2	nd
**11**	8	74 ± 4	65 ± 3	45 ± 4	nd
**12**	3	33 ± 9	14 ± 1	18 ± 3	11 ± 3
**13**	6	21 ± 6	10 ± 3	15 ± 1	12 ± 1
**14**	12	14 ± 1	14 ± 2	16 ± 1	12.1 ± 0.6
**15**	3	21 ± 4	19 ± 2	nd	25 ± 1
**16**	6	13.6 ± 0.6	13 ± 4	nd	12 ± 2
**17**	12	15 ± 1	19 ± 4	nd	12 ± 2
**18**	3	8 ± 1	9 ± 2	8 ± 1	7 ± 1
**19**	12	9 ± 2	15 ± 4	10 ± 2	9 ± 1
**20**	3	>200	>200	>200	>200
**21**	3	>200	>200	>200	>200

nd—not defined. The drug-treatment period was 72 h. The results are expressed as the mean values ± SD from three independent experiments performed in triplicate.

## Data Availability

Data will be made available on request.

## Supplementary Materials

The following supporting information can be downloaded at: https://www.mdpi.com/article/10.3390/pharmaceutics15051366/s1, Figure S1. Experimental spectrum for complex **15**, simulated spectrum for [M + H]^+^, simulated spectrum for [M + Na]^+^; Figure S2. Experimental spectrum for complex **16**, simulated spectrum for [M + H]^+^, simulated spectrum for [M + Na]^+^; Figure S3. Experimental spectrum for complex **17**, simulated spectrum for [M + H]^+^, simulated spectrum for [M + Na]^+^; Figure S4. Experimental spectrum for complex **18**, simulated spectrum for [M − Cl]^+^; Figure S5. Experimental spectrum for complex **19**, simulated spectrum for [M − Cl]^+^; Figure S6. Signal shifts in 1H NMR spectra of ligand **5** and complexes **11**, **14**.
