# Supplementary material for: New Organometallic Ru(II) Compounds with Lonidamine Motif as Antitumor Agents
Source: Pharmaceutics. 2023 Apr 29;15(5):1366. doi: 10.3390/pharmaceutics15051366 (PMC10223346; doi:10.3390/pharmaceutics15051366)
Supplement: Supplementary file 1 [file pharmaceutics-15-01366-s001.zip › pharmaceutics-2313449-supplementary.pdf]

pharmaceutics

Supplementary materials: New Organometallic Ru(II) Compounds with Lonidamine Motif as Antitumor Agents

Ilya A. Shutkov, Yulia N. Okulova, Dmitrii M. Mazur, Nikolai A. Melnichuk, Denis A. Babkov, Elena V. Sokolova, Alexander A. Spasov, Elena R. Milaeva and Alexey A. Nazarov

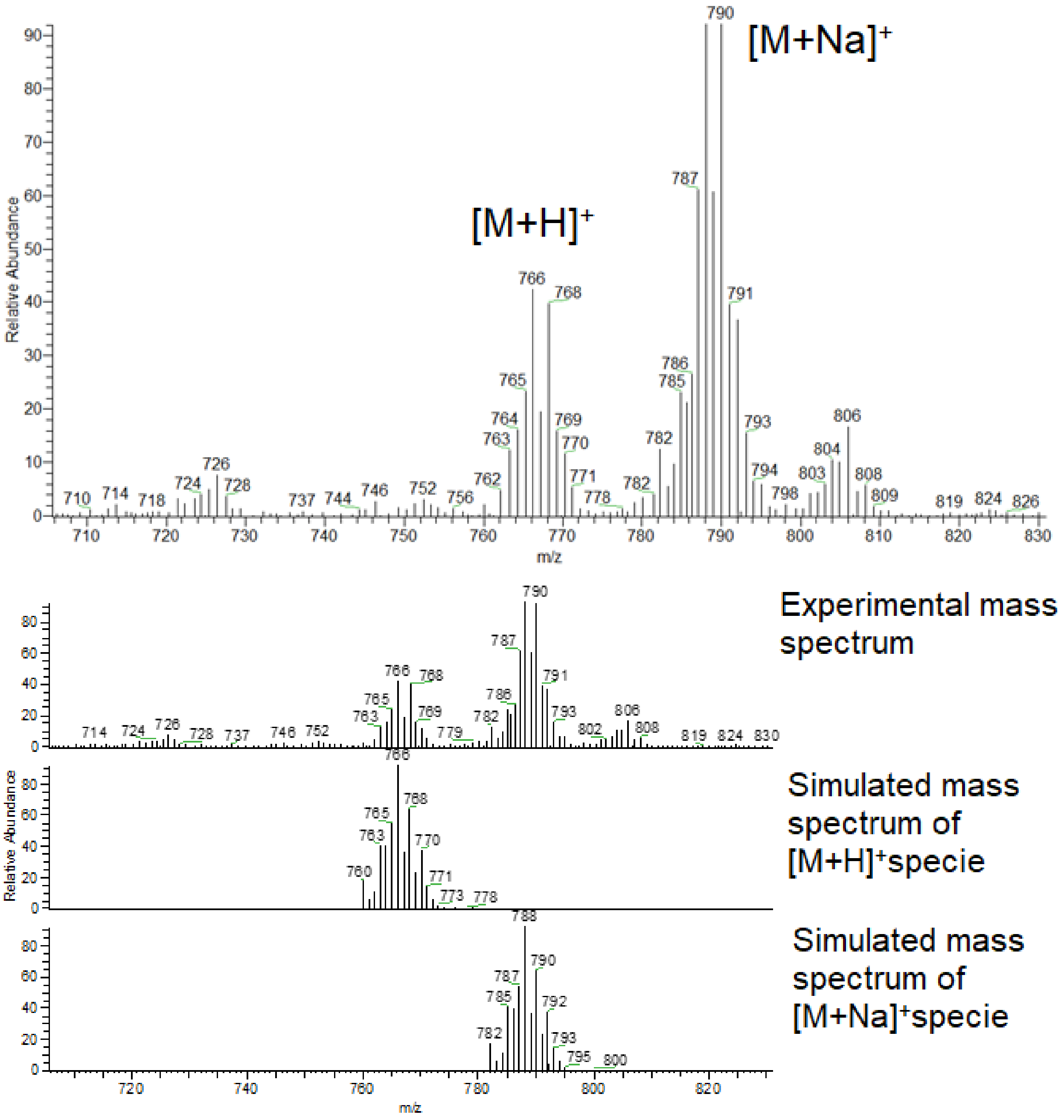

Figure S1. Experimental spectrum for complex **15**, simulated spectrum for $[M+H]^+$, simulated spectrum for $[M+Na]^+$.

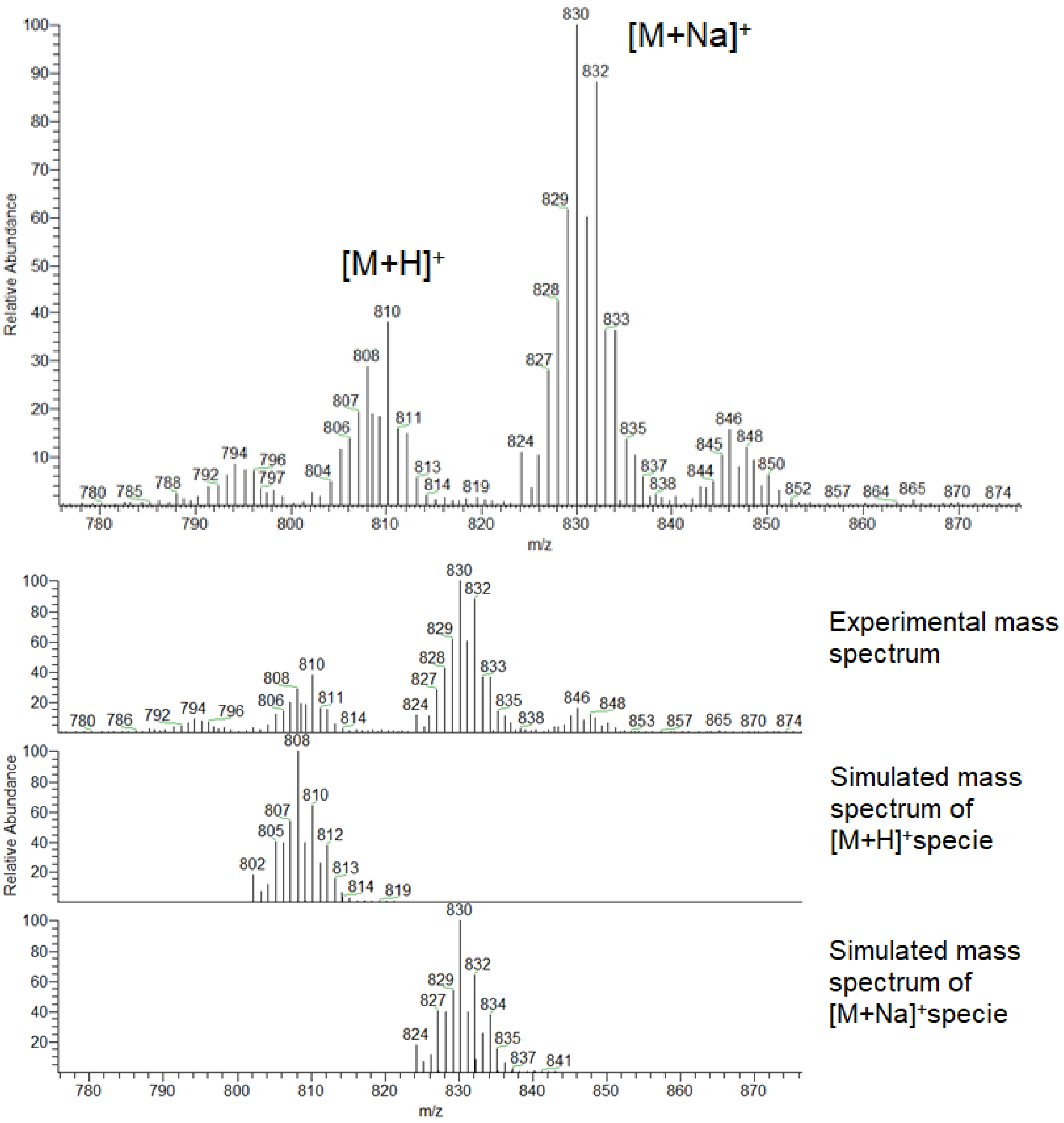

Figure S2. Experimental spectrum for complex **16**, simulated spectrum for $[M+H]^+$, simulated spectrum for $[M+Na]^+$.

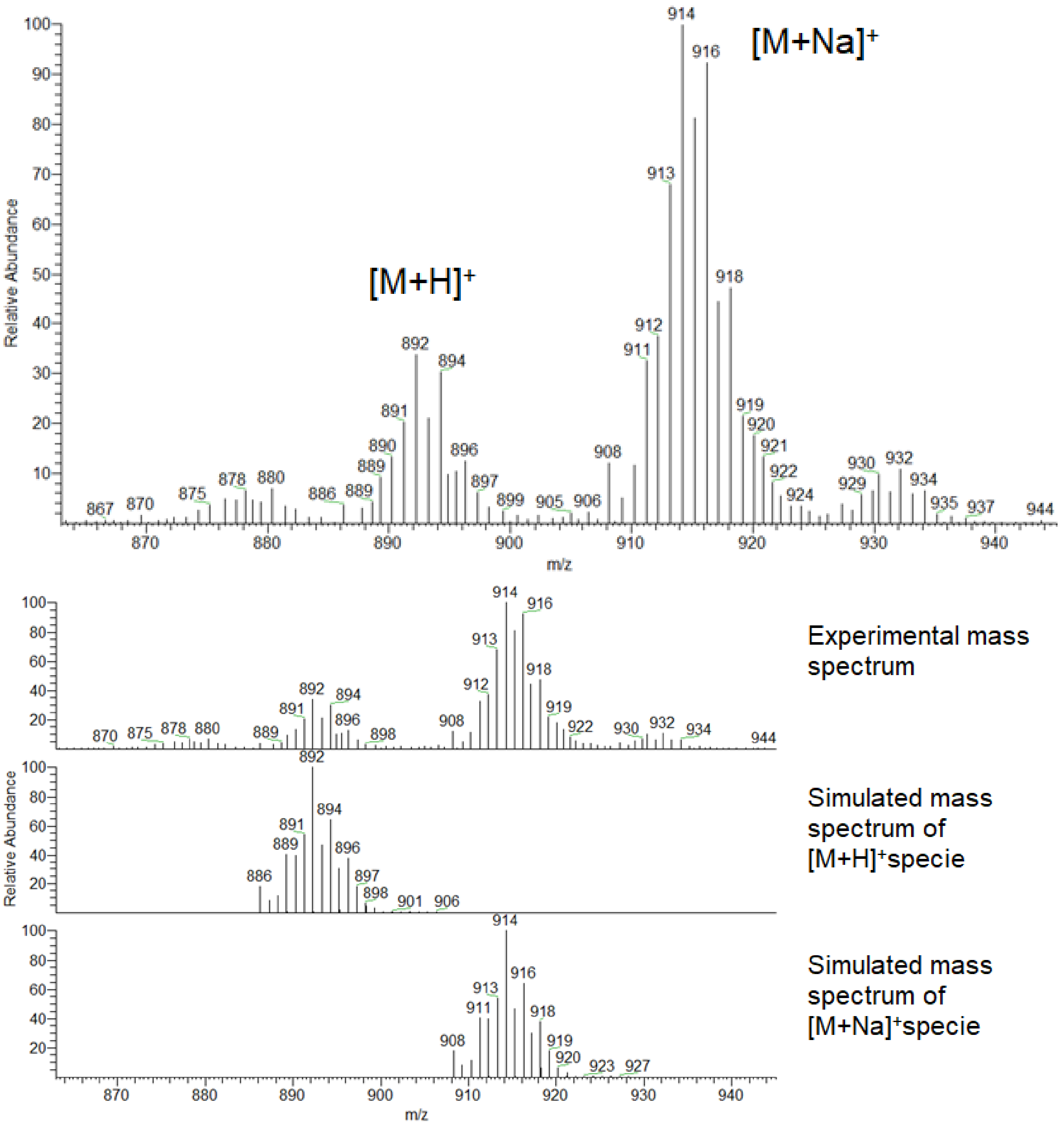

Figure S3. Experimental spectrum for complex **17**, simulated spectrum for $[M+H]^+$, simulated spectrum for $[M+Na]^+$.

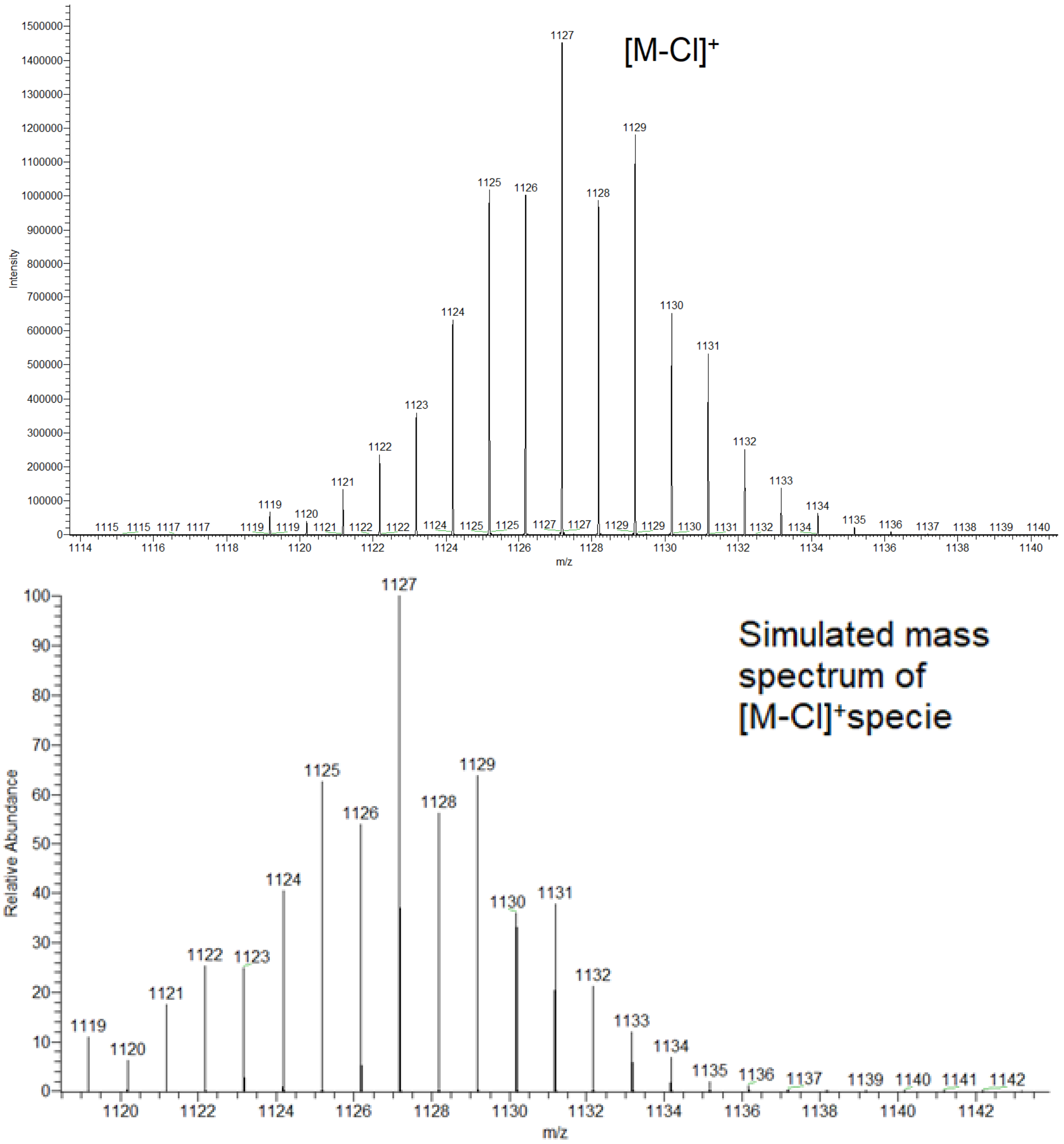

Figure S4. Experimental spectrum for complex **18**, simulated spectrum for $[M\text{-}Cl]^{+}$.

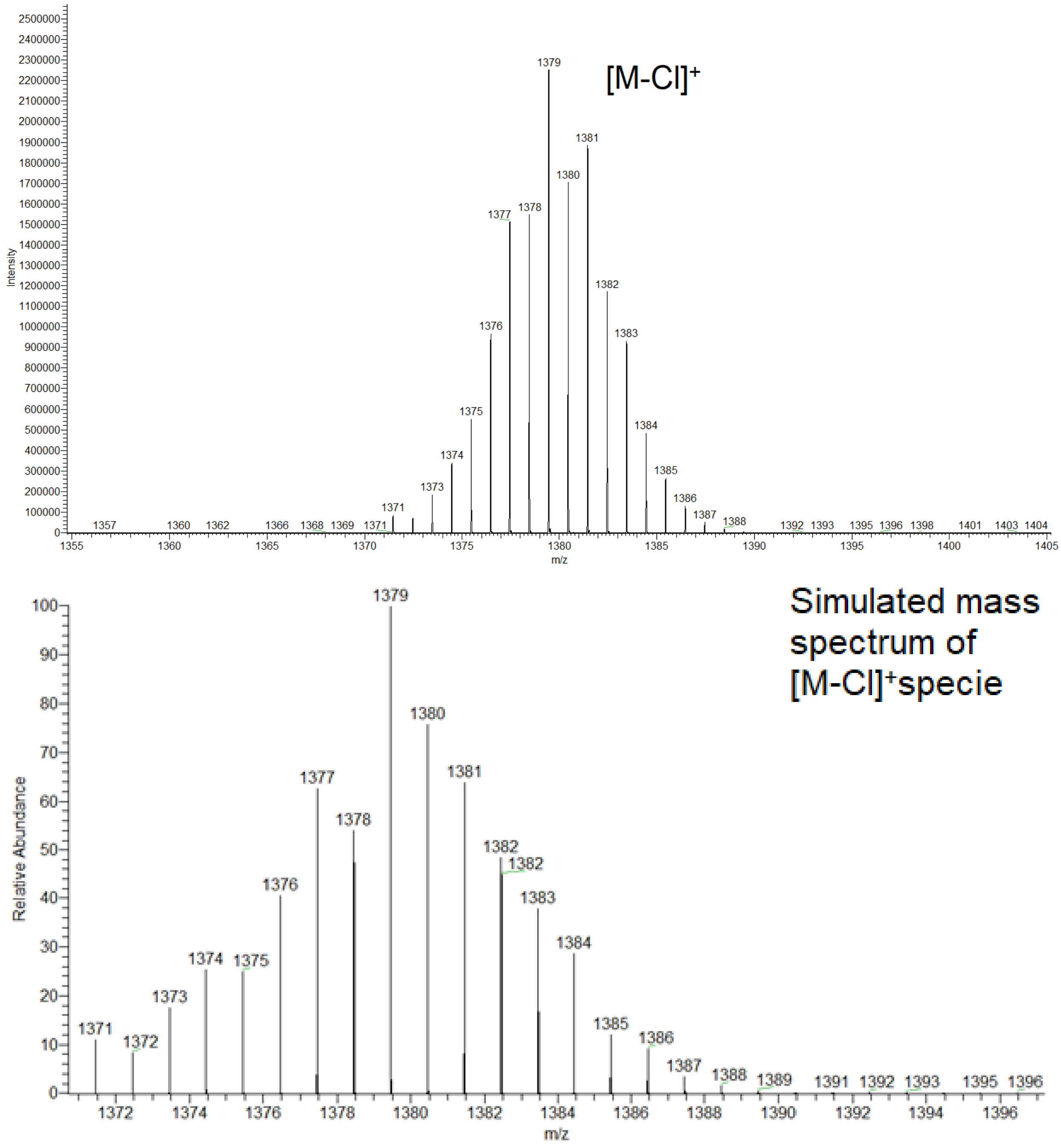

Figure S5. Experimental spectrum for complex **19**, simulated spectrum for [M-Cl]$^{+}$.

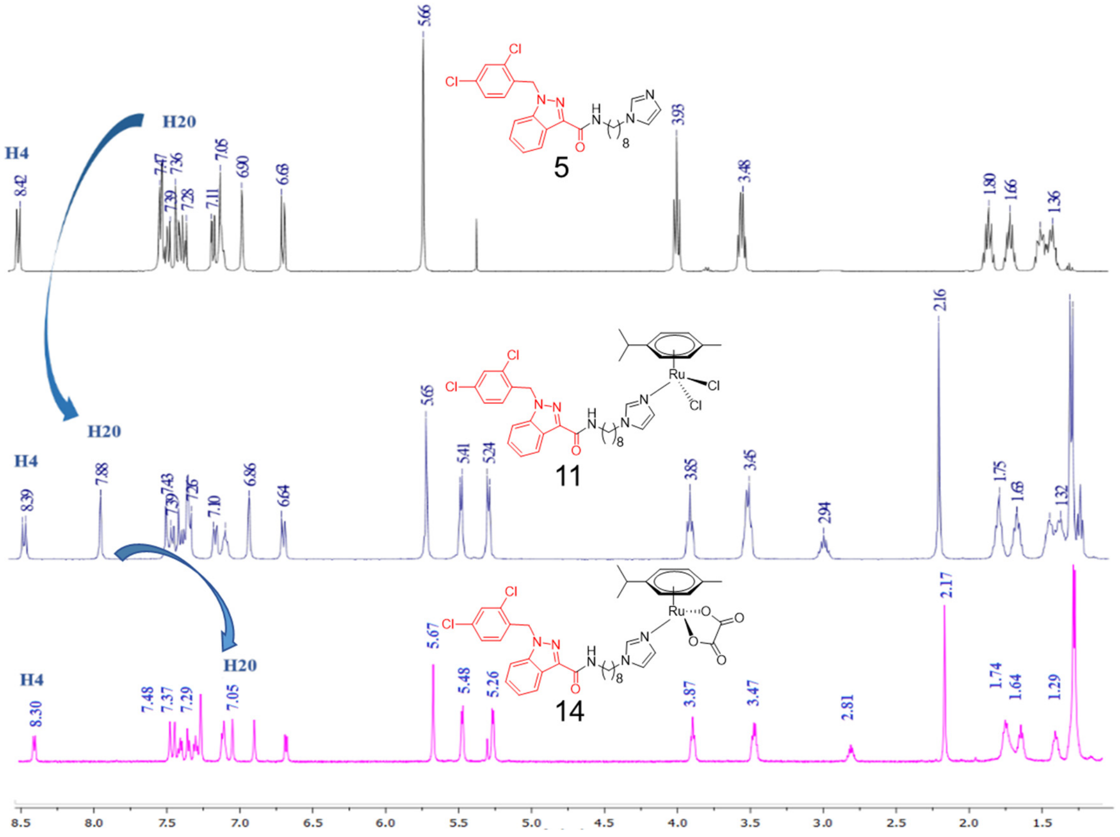

Figure S6. Signal shifts in ^{1}H NMR spectra of ligand **5** and complexes **11**, **14.**
